# Residual Error Coding in NONMEM Can Mislead Diagnostic Residuals: Impact of W Definition on IWRES, WRES, and CWRESI

**DOI:** 10.3390/pharmaceutics18050590

**Published:** 2026-05-10

**Authors:** Nicolas Simon, Katharina von Fabeck

**Affiliations:** Institut de Neurosciences de la Timone, UMR7289 CNRS, Hop Sainte Marguerite, Department of Clinical Pharmacology, CAP-TV, Aix Marseille University, APHM, Marseille, France

**Keywords:** NONMEM, population pharmacokinetics, residual unexplained variability, individual weighted residuals, model diagnostics, $ERROR block

## Abstract

**Background and Objective:** In NONMEM, the residual error model is implemented in the $ERROR block, where the user defines the prediction equation, Y, and a scaling factor, W, used to compute the individual weighted residual. This residual is reported in the diagnostic output as IWRES and corresponds to the individual residual divided by W. The residual error variance entering the likelihood is determined solely by the EPS and SIGMA structure of Y, independently of W. Multiple coding approaches for W are encountered in the literature, but no systematic analysis has examined how these choices affect diagnostic residuals. The aim of this study was to characterize the impact of W coding on three commonly used residual diagnostics in NONMEM, namely, IWRES, WRES, and CWRESI, across additive, proportional, and combined residual error models. **Methods:** Three population pharmacokinetic datasets (500 subjects; 6000 observations each) were simulated from a one-compartment oral model under additive (σ_add = 0.5 mg/L), proportional (CV = 20%), and combined (σ_prop = 0.15, σ_add = 0.5 mg/L) residual error structures. The following nine estimation runs were performed in NONMEM 7.6 (FOCE-I), each differing only in the $ERROR coding of W: normalized SIGMA-based, non-normalized, and THETA-based variants. Diagnostic residuals were compared pairwise by examining observation-by-observation ratios, standard deviations, and Pearson correlations. **Results:** For additive and proportional models, non-normalized W coding produced IWRES compressed by a constant multiplicative factor equal to sqrt(SIGMA(1,1)), reducing SD(IWRES) from 0.933 to 0.269 for the proportional model, while leaving WRES and CWRESI entirely unaffected. THETA-based normalized codings produced IWRES equivalent to SIGMA-based normalized codings. For the combined model, all three coding variants produced similar IWRES, but CWRESI differed by up to 0.586 units between the two-EPS (VAR.1) and one-EPS parameterizations, reflecting differences in NONMEM’s internal variance–covariance matrix structure. The SD coding additionally produced 19 extreme IWRES values (range: −59 to +74) at low predicted concentrations, attributable to the linear approximation of the combined standard deviation. **Conclusions:** The coding of W in NONMEM substantially affects IWRES but not WRES or CWRESI for simple error models. Cross-run comparisons of IWRES are invalid when W is not consistently normalized. For the combined model, the two-EPS VAR.1 parameterization is recommended for population-level diagnostics. These findings provide a practical framework for consistent and interpretable residual error coding in NONMEM.

## 1. Introduction

Residual unexplained variability (RUV) modeling is a fundamental component of population pharmacokinetic (PK) analysis. The residual error model accounts for all sources of variability not captured by structural and statistical models, including assay error, model misspecification, and intra-individual variability [1]. The choice of residual error structure (additive, proportional, or combined) affects not only the precision of parameter estimates but also the validity of model diagnostics and the reliability of simulation outputs. In NONMEM, the residual error model is implemented in the $ERROR block, where the analyst defines the predicted observation (Y), the individual residual (IRES), and a scaling factor (W) used to compute the individual weighted residual (IWRES = IRES/W) [2]. While the mathematical foundations of these models are well established, the practical implementation in NONMEM allows for multiple coding approaches that, although structurally equivalent in terms of the likelihood function and parameter estimation, may differ substantially in their impact on diagnostic residuals.

For IWRES to be interpretable as a standardized residual (approximately normally distributed with a mean of zero and unit variance), W must reflect the true standard deviation of the residual error as encoded in the fitted model [3,4,5,6]. In the remainder of this paper, we refer to such coding as normalized W coding.

For an additive model, this requires: W = SQRT(SIGMA(1,1))

For a proportional model: W = SQRT(IPRED^2^ × SIGMA(1,1))

and for a combined model: W = SQRT(IPRED^2^ × SIGMA(1,1) + SIGMA(2,2))

By contrast, we use the term non-normalized W coding when W omits one or more estimated variance components and, therefore, does not standardize IWRES, even though the underlying likelihood may remain unchanged.

However, alternative coding approaches are also commonly encountered in practice, practical examples, user documentation, and educational contexts, including W = 1 for additive models and W = F or W = IPRED for proportional models [7,8]. Although such codings may be operationally convenient, they do not necessarily standardize IWRES. In these cases, W does not incorporate the estimated variance components, and IWRES is, therefore, not a standardized residual, even though the underlying model fit is identical.

The consequences of different coding choices for the combined residual error model on parameter estimation have been systematically examined by Proost [9]. That study compared three main implementation strategies in NONMEM: the variance-based method (VAR), in which W is defined as the square root of the total residual variance, and the standard-deviation-based method (SD), in which W is expressed as a linear combination of proportional and additive standard deviation terms. Proost demonstrated that different implementations of the VAR method yield identical parameter estimates and OFV, whereas the SD method produces numerically different but statistically valid estimates with a different parameterization of the residual error components [9]. Crucially, however, that analysis was limited to parameter estimation and objective function values. The impact of these coding choices on diagnostic residuals, specifically IWRES, WRES, and CWRESI was not examined.

To our knowledge, no systematic analysis has characterized how the definition of W in the $ERROR block affects the numerical values and diagnostic interpretability of IWRES across additive, proportional, and combined residual error models, nor whether inconsistent W coding across model runs can produce misleading diagnostic plots. This issue may have remained underappreciated because different codings can produce identical likelihoods, objective function values, and parameter estimates in simple settings, while still altering the scale of commonly inspected individual residual diagnostics.

This gap has practical consequences. Although IWRES is no longer the most robust standalone diagnostic in contemporary pharmacometrics, it remains widely reported and visually inspected in routine NONMEM workflows. A pharmacometrician comparing IWRES-based plots between two models coded differently may observe apparent differences in residual dispersion, heteroscedasticity, or concentration-dependent bias that are purely artifactual. This may lead to incorrect conclusions about residual behavior, residual error structure, or perceived model adequacy, even when the underlying fit is unchanged.

The aim of this paper is to provide a systematic and practical characterization of the impact of W coding on diagnostic residuals in NONMEM. The present analysis is therefore intentionally restricted to the NONMEM framework, where the flexibility of the $ERROR block and the implementation of residual diagnostics make this question especially relevant. Using three simulated population PK datasets, each generated under a known additive, proportional, or combined residual error structure, we estimated each model using multiple coding variants of the $ERROR block and compared the resulting IWRES, WRES, and CWRESI values across runs. We illustrate how non-normalized W definitions distort individual-level diagnostic plots, how the choice between one- and two-EPS parameterizations affects population-level residuals in the combined model, and how cross-run comparisons of IWRES can be misleading when W is not consistently defined. Based on these results, we provide practical recommendations for consistent, interpretable residual error coding in NONMEM.

## 2. Methods

### 2.1. Simulation of Population Pharmacokinetic Datasets

Three population PK datasets were generated by stochastic simulation using the rxode2 package (version 5.0.1) in R (version 4.5.1), each corresponding to a distinct residual error structure, as follows: additive, proportional, and combined. A fixed random seed was used throughout to ensure reproducibility. All three datasets were simulated from a one-compartment model with first-order oral absorption, parameterized in terms of apparent clearance (CL/F), apparent volume of distribution (V/F), and absorption rate constant (KA). Between-subject variability (BSV) was implemented on CL/F and V/F using log-normal distributions with a variance of 0.09 on the log scale, corresponding to an approximate coefficient of variation of 30% for each parameter. This level of variability was chosen as a moderate and realistic didactic setting, sufficient to generate plausible interindividual PK differences while preserving a clear interpretation of the residual error coding effects under study.

For each dataset, 500 subjects received a single oral dose of 100 mg, with 12 concentration measurements collected per subject at nominal times of 0.25, 0.5, 1, 1.5, 2, 4, 6, 8, 10, 12, 18, and 24 h post-dose, yielding 6000 observations per dataset. The true population parameters used for simulation were TVCL = 5.0 L/h, TVV = 50.0 L, and KA = 1.0 h^−1^ (fixed). Residual error was added to the individual model-predicted concentrations (IPRED) according to the structure of each dataset. For the additive dataset, a single normally distributed error term was used with true standard deviation σ_add = 0.5 mg/L. For the proportional dataset, a multiplicative error was applied with true proportional coefficient of variation CV = 20% (σ_prop = 0.20). For the combined dataset, two independent error terms were used simultaneously, a proportional component with σ_prop = 0.15 and an additive component with σ_add = 0.5 mg/L, consistent with the structure described by Proost [9]. These values were selected to create three distinct and interpretable residual error scenarios. The proportional-only dataset used a slightly larger proportional component to represent a clearly multiplicative error structure, whereas the combined dataset used a somewhat smaller proportional contribution so that both additive and proportional variability would remain visible across the concentration range. Simulated concentrations below 0.001 mg/L were truncated to that value to avoid numerical issues. All true simulation parameter values are summarized in Table 1.

### 2.2. Estimation Models

Each dataset was analyzed in NONMEM (version 7.6; Icon Development Solutions, Ellicott City, MD, USA) using the first-order conditional estimation method with the INTERACTION option (FOCE-I; “METHOD = 1 INTERACTION”). The structural model and between-subject variability structure were identical across all runs and matched the true simulation model, as follows: a one-compartment model with first-order absorption (ADVAN2 TRANS2), with BSV on CL and V modeled as exponential random effects. KA was fixed to its true simulation value of 1.0 h^−1^ in all runs to focus comparisons exclusively on residual error behavior. The following nine estimation runs were performed in total, grouped by residual error structure:

**Additive error models (applied to the additive dataset):**

- ADD.1: normalized coding: “W = SQRT(SIGMA(1,1))”, “Y = IPRED + ERR(1)”. The scaling factor W explicitly incorporates the estimated residual standard deviation, yielding a properly standardized IWRES;
- ADD.2: non-normalized coding: “W = 1”, “Y = IPRED + W*ERR(1)”. Although the Y equation is structurally identical to ADD.1 and, therefore, produces the same likelihood, OFV, and parameter estimates, W does not incorporate the estimated variance component, resulting in an unnormalized IWRES;
- ADD.3: THETA-based normalized coding: “W = THETA(4)”, “Y = IPRED + W*ERR(1)”, with “$SIGMA 1 FIX”. The residual standard deviation is estimated as a fixed effect, with SIGMA fixed to unity. This parameterization is expected to yield a normalized IWRES numerically equivalent to ADD.1 under correct model convergence.

**Proportional error models (applied to the proportional dataset):**

- PROP.1: normalized coding: “W = SQRT(IPRED**2 * SIGMA(1,1))”, “Y = IPRED + IPRED*ERR(1)”. W corresponds to the model-implied observation-specific residual standard deviation;
- PROP.2: non-normalized coding: “W = IPRED”, “Y = IPRED + IPRED*ERR(1)”. Y is structurally identical to PROP.1 and produces the same estimates, but W omits the SIGMA term, yielding an unnormalized IWRES;
- PROP.3: THETA-based normalized coding: “W = IPRED * THETA(4)”, “Y = IPRED + W*ERR(1)”, with “$SIGMA 1 FIX”. THETA(4) estimates the proportional coefficient of variation as a dimensionless fraction.

**Combined error models (applied to the combined dataset):**

- COMB VAR.1: variance-based method with two EPS (Proost VAR.1): “W = SQRT(IPRED**2 * SIGMA(1,1) + SIGMA(2,2))”, “Y = IPRED + IPRED*ERR(1) + ERR(2)”. This is the canonical two-epsilon parameterization, in which SIGMA(1,1) and SIGMA(2,2) represent the proportional and additive variance components, respectively. This coding matches the true simulation structure;
- COMB VAR.3: variance-based method with one EPS (Proost VAR.3): “W = SQRT(IPRED**2 * THETA(4)**2 + THETA(5)**2)”, “Y = IPRED + W*ERR(1)”, with “$SIGMA 1 FIX”. THETA(4) and THETA(5) estimate the proportional and additive standard deviations, respectively, as fixed effects. W is constructed to equal the true residual standard deviation of the combined model;
- COMB SD: standard-deviation-based method (Proost SD): “W = THETA(4)*IPRED + THETA(5)”, “Y = IPRED + W*ERR(1)”, with “$SIGMA 1 FIX”. As discussed by Proost (2017), this W does not represent the true standard deviation of the combined variance model but a linear approximation thereof, and it yields different but statistically valid parameter estimates compared to the VAR methods.

The nomenclature for COMB VAR.1, COMB VAR.3, and COMB SD follows that of Proost (2017) [9] to facilitate cross-referencing with that work.

### 2.3. Comparison of Diagnostic Residuals

For each estimation run, the NONMEM output table (“.fit”) was generated to include the following variables: “ID”, “TIME”, “DV”, “IPRED”, “IWRES”, “WRES”, “CWRESI”, “PRED”, and “RES”. All post-processing and comparisons were performed in R (version 4.5.1).

Pairwise comparisons of IWRES, WRES, and CWRESI were conducted between runs sharing the same dataset and differing only in their $ERROR coding. These comparisons were based on both numerical summaries and graphical diagnostics, so that the conclusions of the study would not rely on visual inspection alone. For each pair, the following three numerical metrics were computed: (i) the observation-by-observation ratio of IWRES values, summarized as the mean ± standard deviation across all observations; (ii) the Pearson correlation coefficient between corresponding residual vectors; and (iii) the standard deviation of IWRES across all observations, used as a measure of correct normalization. A well-specified model with a correctly normalized W is expected to yield an SD(IWRES) close to 1.0 [5].

For the non-normalized coding schemes (ADD.2 and PROP.2), the implicit effective scaling factor, that is, the value of W that would be required to reproduce the same IWRES as the normalized reference run, was back-calculated observation by observation as the ratio of the IRES to IWRES from the non-normalized run and compared to the theoretical expectation derived from the estimated SIGMA of the corresponding reference run.

### 2.4. Evaluation of Diagnostic Plots

Graphical diagnostics were generated for each run using ggplot2 4.0.3 in R. To illustrate the impact of W coding on individual-level diagnostics, IWRES versus IPRED plots were constructed for the normalized and non-normalized coding variants within each error type. For the combined model, CWRESI versus IPRED plots were compared across coding variants rather than IWRES, because it is the population-level residual that differs meaningfully across combined error parameterizations. Finally, a cross-model scenario was constructed by superimposing IWRES versus IPRED plots from a non-normalized additive run and a normalized proportional run, to illustrate how inconsistent W definitions across runs can produce apparently meaningful differences in residual scatter that are purely artifactual.

### 2.5. Code Availability

All NONMEM control files (.ctl) corresponding to the coding variants analyzed in the manuscript, the rxode2 simulation script, and the R analysis scripts used to generate the figures and tables are provided as Supplementary Materials. The simulated datasets (additive, proportional, and combined) are also included to allow for full reproduction of the estimation results. The Supplementary Materials is intended not only to support reproducibility but also to provide directly reusable examples of the alternative $ERROR codings discussed in the main text.

### 2.6. Language Editing and Stylistic Improvements

The authors used a generative AI tool (ChatGPT 5.5) to assist with language editing and stylistic improvements. The scientific content, analyses, and interpretations were fully developed and verified by the authors, who take full responsibility for the manuscript.

## 3. Results

### 3.1. Dataset Structure

All nine estimation runs converged successfully. Each (*.fit) output file contained 6000 observations from 500 subjects, confirming that no observations were lost during the truncation of simulated concentrations. The three datasets were structurally identical in terms of design, differing only in the residual error structure used for simulation.

### 3.2. Additive Residual Error Model

#### 3.2.1. IWRES

ADD.1 and ADD.2 produced identical parameter estimates and OFV because they define the same observation model Y and, therefore, the same likelihood. The difference lies only in the scaling factor W used to compute IWRES, which changes residual diagnostics but not the fit itself.

The three additive coding variants produced markedly different IWRES distributions despite being fitted to the same dataset. For ADD.1 (normalized reference, W = SQRT(SIGMA(1,1))), the IWRES had a mean of 0.006 and a standard deviation of 0.947 (Table 2 (Panel B)).

For ADD.2 (non-normalized, W = 1), the mean was 0.003 and the standard deviation was 0.44. This indicates that IWRES are artificially compressed when W does not incorporate the residual standard deviation, despite the underlying model fit being identical (Table 2 (Panel B)). For ADD.3 (THETA-based, W = THETA(4)), the mean was 0.005 and the standard deviation was 0.947, identical to ADD.1 and close to the theoretically expected value of 1.0 (Table 2 (Panel B)).

The pairwise ratio ADD.2/ADD.1 had a mean of 0.465 with a standard deviation of 0.002, confirming that the IWRES values from the two runs are proportional but not identical. The small variability in the ratio reflects minor differences in IPRED arising from the slightly different parameter estimates between the two runs, despite their structurally identical Y equations. The Pearson correlation between IWRES vectors was 1.000 in both cases, confirming that the rank ordering of residuals is perfectly preserved across all three codings.

The non-constant ratio for ADD.2 relative to ADD.1 deserves attention. Algebraically, if IPRED values were perfectly identical between runs, IWRES_ADD2/IWRES_ADD1 = sqrt(SIGMA(1,1)) would be a true constant. In practice, the maximum difference in IPRED values between ADD.1 and ADD.2 was 1 × 10^−4^ mg/L, numerically negligible, yet the ratio shows a small but detectable spread. This reflects the fact that even when the Y equations are structurally identical, minor numerical differences in the optimization path can produce marginally different parameter estimates, which propagate to the IPRED and then IRES.

The back-calculated implicit W for ADD.2 had a mean of 1.000 (SD = 0.012), confirming that W = 1 was correctly applied as coded. For ADD.3, the implicit W had a mean of 0.465 (SD = 0.006), consistent with the THETA(4) estimate obtained by NONMEM for the additive standard deviation in this run.

#### 3.2.2. WRES and CWRESI

WRES and CWRESI were numerically indistinguishable across all three additive coding variants. The maximum absolute difference between ADD.1 and ADD.2 was 1 × 10^−4^ for both WRES and CWRESI, and similarly for ADD.1 versus ADD.3. These differences are attributable to NONMEM’s internal rounding in output tables and are not analytically meaningful. The standard deviation of CWRESI was 1.000 for all three runs, consistent with the expected unit-variance property of this population-level residual.

**Figure 1:**
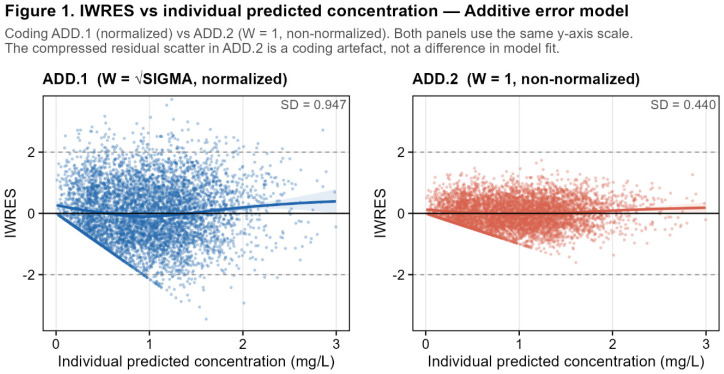
Individual weighted residuals (IWRES) versus individual predicted concentrations (IPRED) for the additive residual error model: (**left panel**) normalized coding ADD.1 (W = SQRT(SIGMA(1,1))); (**right panel**) non-normalized coding ADD.2 (W = 1). Both runs were fitted to the same simulated dataset (n = 6000 observations; 500 subjects) and produced identical parameter estimates and objective function values. The compressed scale of IWRES in ADD.2 (SD = 0.44 vs. 0.95 for ADD.1) is a direct consequence of the non-normalized W and does not reflect a difference in model fit. Take-home message: for additive models, W coding can strongly alter IWRES appearance while leaving model fit and population residuals unchanged.

The coding of W has a substantial impact on IWRES but leaves WRES and CWRESI entirely unaffected. Among the three variants, both ADD.1 and ADD.3 produce IWRES with a standard deviation close to 1.0 (0.947 for both), confirming that the two normalized codings are equivalent in terms of residual standardization, while ADD.2 yields SD(IWRES) = 0.440. Diagnostic plots based on IWRES will, therefore, display very different scales of residual scatter between the normalized and non-normalized runs, even though all three represent the same underlying model fit (Figure 1).

### 3.3. Proportional Residual Error Model

#### 3.3.1. IWRES

The proportional model results were the most analytically clean of the three error types. PROP.1 (normalized, W = SQRT(IPRED^2^ × SIGMA(1,1))) and PROP.3 (THETA-based, W = IPRED × THETA(4)) produced strictly identical IWRES distributions: mean = 0.037, SD = 0.933 for both, with a pairwise ratio mean of 1.000 (SD = 0.007) and Pearson r = 1.000. This confirms that PROP.3 is a true normalized parameterization equivalent to PROP.1 in terms of residual standardization.

PROP.2 produced a markedly compressed IWRES distribution (SD = 0.27). This confirms that non-normalized W coding rescales IWRES by a constant multiplicative factor without affecting the underlying model fit. The pairwise ratio PROP.2/PROP.1 was essentially constant across all 6000 observations (mean = 0.288, SD = 5.6 × 10^−6^, min = 0.288, max = 0.29). This near-perfect constancy arises because, for the proportional model, PROP.1 and PROP.2 share identical Y equations (Y = IPRED + IPRED * ERR(1)) and, therefore, produce rigorously identical IPRED values (maximum IPRED difference = 0). Consequently, IRES is identical across the two runs, and the ratio reduces exactly to the following:

IWRES_PROP.2_/IWRES_PROP.1_ = (IRES/IPRED)/{IRES/(IPRED * SQRT(SIGMA(1,1)))} = SQRT(SIGMA(1,1))

The observed constant ratio of 0.288, therefore, implies an estimated SIGMA(1,1) of 0.08 for this run, corresponding to a proportional CV of approximately 28.8%, reflecting the specific realization of the simulated dataset and the estimation of both structural and variability parameters.

The estimated proportional CV of 28.8%, rather than the simulated 20%, was observed consistently across the proportional model codings. This difference, therefore, cannot be attributed to the normalized versus non-normalized W coding alone but rather reflects estimation on this simulated dataset and/or the specific parameterization used.

#### 3.3.2. WRES and CWRESI

WRES and CWRESI were rigorously identical between PROP.1 and PROP.2 (maximum absolute difference = 0 for both), and essentially identical between PROP.1 and PROP.3 (maximum absolute difference ≤9 × 10^−4^ for WRES, ≤3 × 10^−4^ for CWRESI), with these negligible differences attributable to output rounding. The SD of CWRESI was 1.009 across all three proportional runs.

**Figure 2:**
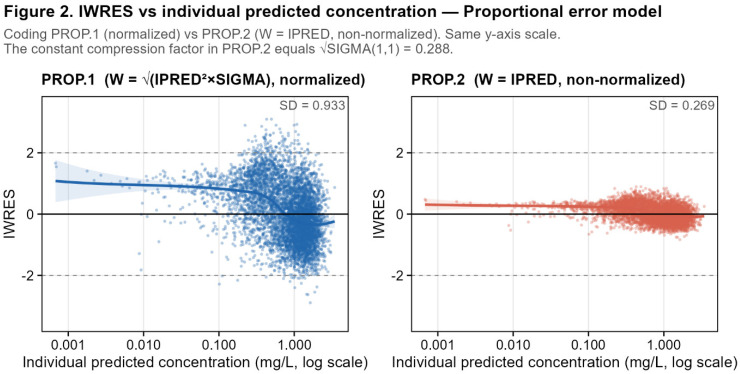
Individual weighted residuals (IWRES) versus individual predicted concentrations (IPRED, log scale) for the proportional residual error model: (**left panel**) normalized coding PROP.1 (W = SQRT(IPRED^2^ × SIGMA(1,1))); (**right panel**) non-normalized coding PROP.2 (W = IPRED). Both runs produced rigorously identical IPRED values and a perfectly constant IWRES ratio of 0.2880 across all observations. The three-fold compression of residual scatter in PROP.2 (SD = 0.27 vs. 0.93 for PROP.1) is entirely attributable to the omission of SQRT(SIGMA(1,1)) from W. Take-home message: in proportional models, non-normalized W produces a visually misleading compression of IWRES despite identical fitted predictions.

The non-normalized coding PROP.2 compresses IWRES by a factor equal to sqrt(SIGMA(1,1)), a perfectly constant multiplicative factor, while leaving WRES and CWRESI completely unaffected. The constancy of this ratio in the proportional case, in contrast to the near-constancy observed in the additive case, directly reflects whether the structural model predictions are identical between runs. For the proportional error model, because W does not enter the likelihood when structured as Y = IPRED + IPRED * ERR(1), the parameter estimates, and therefore IPRED, are rigorously identical across PROP.1 and PROP.2 (Figure 2).

### 3.4. Combined Residual Error Model

Although the models remained structurally comparable, the recovery of the simulated residual error parameters was not exact in all parameterizations, especially for the proportional and combined models.

In the combined residual error models, an important conceptual distinction must be made between variance-based (VAR) parameterizations, which reflect the true variance structure of the model, and standard-deviation-based (SD) parameterizations, which rely on a linear approximation of the residual standard deviation.

#### 3.4.1. IWRES

The three combined error coding variants produced highly concordant IWRES distributions. COMB VAR.1 and COMB VAR.3 yielded virtually identical IWRES: mean = 0.02 and SD = 0.95 for both, with a pairwise ratio mean of 1.00 (SD = 0.003, min = 0.86, max = 1.06) and Pearson r = 1.000. This confirms that the VAR.1 and VAR.3 parameterizations are equivalent in terms of individual-level residual standardization, in agreement with the parameter estimation equivalence reported by Proost (2017) [9].

COMB SD also produced an IWRES closely aligned with COMB VAR.1 at the population level (mean = 0.02, SD = 0.95, Pearson r = 0.999), but the pairwise ratio showed substantial instability, as follows: mean = 0.998, SD = 1.61, with extreme values ranging from −59.0 to +74.3. These 19 extreme observations do not reflect model misspecification but rather a numerical artifact of the SD parameterization. At low predicted concentrations, the linear approximation of the standard deviation diverges from the true variance structure, leading to unstable IWRES values.

#### 3.4.2. WRES and CWRESI

Unlike the additive and proportional cases, the combined error model showed non-negligible differences in WRES and CWRESI across the coding variants, reflecting the impact of the one- versus two-EPS structures and the SD approximation on NONMEM’s internal variance–covariance matrix computation.

COMB VAR.1 versus COMB VAR.3: WRES differed by up to 0.039 (mean absolute difference = 0.006), and CWRESI differed by up to 0.586 (mean absolute difference = 0.01). Despite these differences, the Pearson correlations were 1.000 for WRES and 0.9998 for CWRESI, confirming high concordance with small systematic deviations attributable to the different internal representation of the residual variance matrix in the one-EPS versus two-EPS parameterizations.

COMB VAR.1 versus COMB SD: WRES differed by up to 0.35 (mean = 0.02) and CWRESI by up to 0.58 (mean = 0.02). The Pearson correlations remained high (0.9995 for WRES and 0.9995 for CWRESI), but the larger maximum differences reflect the structural divergence between the SD approximation and the true variance model, particularly at the extremes of the concentration range. The SD of CWRESI was 1.003 for VAR.1, 1.02 for VAR.3, and 1.01 for COMB SD, all within an acceptable range for a well-specified model.

**Figure 3:**
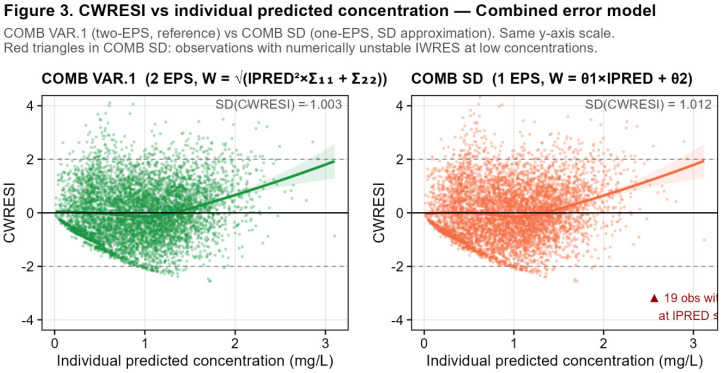
Conditional weighted residuals (CWRESI) vs. individual predicted concentrations (IPRED) for the combined residual error model: (**left panel**) two-EPS variance-based coding COMB VAR.1; (**right panel**) standard-deviation-based coding COMB SD. Observations with extreme IWRES ratio (|ratio| > 5, n = 19, IPRED ≤ 1.94 mg/L) are highlighted as open triangles. These extreme values arise from the linear approximation of the combined standard deviation in COMB SD at low predicted concentrations, where W = THETA(4) × IPRED + THETA(5) diverges from the true standard deviation SQRT(IPRED^2^ × σ^2^prop + σ^2^add). Horizontal dashed lines at CWRESI = ±2. Blue: COMB VAR.1; red-orange: COMB SD. CWRESI is shown rather than IWRES because, unlike for the additive and proportional models, it is the population-level residual that differs meaningfully across combined error coding variants. Take-home message: in combined models, coding choices can affect population residuals even when individual residual behavior remains globally similar.

Unlike the additive and proportional cases—where WRES and CWRESI were unaffected by the W coding—the combined error model introduces differences in population-level residuals when moving from the two-EPS structure (VAR.1) to the one-EPS alternatives (VAR.3 and SD). These differences are numerically small on average but can reach nearly 0.6 units in CWRESI for individual observations, and are concentrated in the extreme tails of the residual distribution. For the VAR.3 coding, this arises from NONMEM’s different internal handling of the variance–covariance structure with a single EPS; for COMB SD, it reflects the additional approximation error of the linear SD model at low concentrations (Figure 3).

### 3.5. Cross-Model Scenario: Risk of Misleading Comparison

The analytical results above demonstrate a practical hazard that arises when IWRES-based diagnostic plots are compared across runs that use different W definitions. To illustrate this, consider a pharmacometrician comparing an additive model coded as ADD.2 (W = 1, non-normalized) with a proportional model coded as PROP.1 (W normalized). The SD(IWRES) for ADD.2 is 0.44, while for PROP.1 it is 0.93, a more than two-fold difference. A visual comparison of IWRES vs. IPRED plots between these two runs would suggest that the proportional model has substantially larger and more dispersed residuals than the additive model. If interpreted in isolation, this could be mistaken for evidence of a worse fit or model misspecification, even though formal model evaluation should rely on multiple complementary criteria. In reality, this difference is purely an artifact of the inconsistent normalization: ADD.2 compresses its IWRES by a factor of approximately 0.465 relative to a properly normalized additive run, while PROP.1 correctly standardizes its residuals to unit variance. The underlying model fits, as captured by WRES, CWRESI, and OFV, are entirely unrelated to this visual discrepancy.

This scenario illustrates that cross-run comparisons of IWRES are only valid when W is consistently defined as the true standard deviation of the residual error model in all runs being compared.

**Figure 4:**
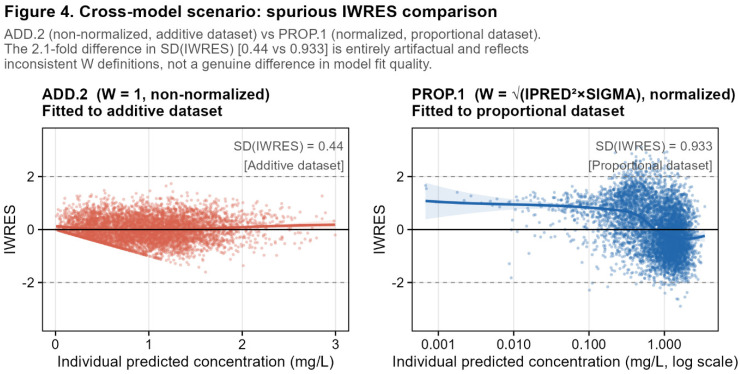
Cross-model comparison illustrating the risk of spurious inference from inconsistent W coding. IWRES vs. IPRED for ADD.2 (additive dataset, non-normalized coding, W = 1; red-orange) overlaid with PROP.1 (proportional dataset, normalized coding; blue). The apparent two-fold difference in residual scatter (SD(IWRES) = 0.44 for ADD.2 versus 0.93 for PROP.1) is entirely artifactual: it reflects the compression introduced by non-normalized W in ADD.2, not a genuine difference in model adequacy, residual variability, or fit quality between the additive and proportional models. WRES and CWRESI are unaffected by this coding difference. Horizontal dashed lines indicate IWRES = ±2. Take-home message: IWRES plots should not be compared across runs unless W is defined consistently as the model-implied residual standard deviation.

In the absence of this consistency, IWRES-based plots should not be interpreted as direct evidence of relative fit quality (Figure 4). Model evaluation should, instead, remain based on a broader set of criteria, including OFV, CWRESI, simulation-based diagnostics, clinical plausibility, and the intended use of the model.

## 4. Discussion

### 4.1. Principal Findings

This study provides the first systematic numerical characterization of the impact of W coding in the NONMEM $ERROR block on diagnostic residuals, across additive, proportional, and combined residual error models. Three principal findings emerge.

**First**, for additive and proportional models, the definition of W has a direct, quantifiable, and algebraically predictable impact on IWRES, while leaving WRES and CWRESI strictly unchanged. This effect is multiplicative and proportional to the estimated variance component. For the additive model, ADD.1 (W = SQRT(SIGMA(1,1))) estimated SIGMA(1,1) = 0.216, giving W = 0.465. ADD.2 (W = 1), despite sharing a structurally identical Y equation, estimated SIGMA(1,1) = 0.22, producing SD(IWRES) = 0.44. The ratio SD(IWRES_2_)/SD(IWRES_1_) = 0.47 equals √(SIGMA_1_) = √0.22, confirming the algebraic relationship. ADD.3, with W = THETA(4) = 0.465 and $SIGMA 1 FIX, produced a normalized IWRES with SD = 0.947, identical to ADD.1, confirming the equivalence of SIGMA-based and THETA-based normalized codings.

This confirms that the difference between these codings concerns residual standardization rather than model fit. The mechanistic basis of this point is developed in Section 4.2.

**Second**, for the proportional model, the result is even more striking. PROP.1 and PROP.2 produced rigorously identical parameter estimates (SIGMA(1,1) = 0.083, CL = 4.72 L/h, V = 34.8 L, OFV = −9622.81), and the pairwise ratio IWRES_2_/IWRES_1_ was perfectly constant at 0.288 (SD = 5.6 × 10^−6^) (Table 2 (Panel A)). This near-exact constancy arises because the two runs share a structurally identical Y = IPRED + IPRED*ERR(1) equation, which produces rigorously identical IPRED values (maximum difference = 0). Consequently, IRES is identical across both runs, and the compression factor reduces exactly to √(SIGMA(1,1)) = √0.082931 = 0.288.

**Third**, for the combined error model, all three codings (VAR.1, VAR.3, COMB SD) produced globally concordant IWRES (Pearson r ≥ 0.999 for all pairs) and identical OFV for VAR.1 and VAR.3 (−1800.81). However, CWRESI differed non-negligibly between the one-EPS and two-EPS parameterizations (maximum difference of up to 0.586), and COMB SD generated 19 extreme IWRES observations (0.32%) at low predicted concentrations (IPRED ≤ 1.94 mg/L), with values ranging from −59.0 to +74.3.

### 4.2. The Mechanistic Basis of W’s Impact on IWRES

The behavior of IWRES across alternative codings arises from the way residual variability is parameterized in the observation model.

In NONMEM, the likelihood and the objective function value (OFV) are determined by the statistical model defined in the $ERROR block through the equation used to compute Y. The variance entering the likelihood is, therefore, governed by the EPS/SIGMA structure embedded in Y, rather than by the user-defined variable W. Consequently, when different codings produce the same observation model Y, they necessarily produce the same likelihood, identical parameter estimates, and identical OFV values.

This explains the invariance observed between codings such as ADD.1 and ADD.2 or PROP.1 and PROP.2. In these cases, the observation model is unchanged and the residual variance structure entering the likelihood remains identical. The user-defined variable W is used only in the computation of diagnostic residuals through the expression

IWRES = IRES/W

and, therefore, affects the scaling of IWRES without affecting the likelihood or the fitted parameters.

More generally, the quantity represented by W corresponds to the model-predicted standard deviation of the residual error for a given observation. When W is correctly defined as the square root of the residual variance implied by the model, IWRES are expected to behave approximately as standardized normal residuals. When W is defined differently, IWRES are simply rescaled versions of the same individual residuals, which explains the substantial differences in IWRES distributions observed across codings that, nevertheless, produce identical OFV and parameter estimates.

A different situation arises when residual variability is parameterized directly within the definition of Y, for example through THETA-based parameterizations with SIGMA fixed to one. In these cases, the residual variance entering the likelihood is carried by model parameters appearing explicitly in Y. Different codings may, therefore, represent alternative parameterizations of the same variance model, potentially preserving the OFV while modifying the interpretation and numerical values of estimated residual error parameters.

Taken together, these results show that the apparent impact of W coding on IWRES reflects a change in residual standardization rather than a change in model fit. The likelihood remains unaffected as long as the statistical model defined in Y is unchanged.

### 4.3. Why SD(IWRES) ≠ 1.0 for Normalized Runs: ε-Shrinkage

A potentially unexpected finding is that ADD.1, despite being correctly normalized, yields SD(IWRES) = 0.947, below the theoretical value of 1.0. This is consistent with ε-shrinkage [5]. When individual empirical Bayes estimates shrink individual predictions towards the population, IRES are compressed towards zero, reducing SD(IWRES) below 1.0. The quantity 1 − SD(IWRES) estimates the ε-shrinkage: for ADD.1, ε-shrinkage = 1 − 0.947 = 5.3%, indicating modest shrinkage consistent with a well-specified additive model in this dataset. This explains why SD(IWRES) rarely equals exactly 1.0 in real datasets, even when W is correctly defined.

Among normalized codings, ADD.1 and ADD.3 produced identical SD(IWRES) = 0.947, both slightly below the theoretical value of 1.0 due to ε-shrinkage. Despite identical OFV values (−2095.254), the two runs estimated different residual standard deviations in different parameterizations: W = 0.465 for ADD.1 (from SQRT(SIGMA(1,1)) = SQRT(0.216)) versus THETA(4) = 0.465 for ADD.3, with SIGMA fixed to 1. These are numerically equivalent representations of the same residual standard deviation, confirming that THETA-based and SIGMA-based normalized codings produce equivalent IWRES when correctly specified.

For PROP.1, ε-shrinkage = 1 − 0.933 = 6.7%, substantially lower and expected for a proportional model that adapts more naturally to the heteroscedastic data structure. This difference illustrates that ε-shrinkage is a property of the data and model structure, not of the coding.

It is, therefore, useful to distinguish, at a conceptual level, the systematic deviation of SD(IWRES) from 1.0 due to ε-shrinkage from the artificial compression introduced by non-normalized W. In simple settings these two effects can be discussed separately, but in practice they may interact, particularly in combined error models and at low predicted concentrations. Correctly defining W removes the coding-related compression of IWRES, but it does not eliminate shrinkage-related departures from the ideal unit-variance behavior.

### 4.4. Why WRES and CWRESI Are Invariant to W Coding in Simple Models

The invariance of WRES and CWRESI to W coding for the additive and proportional models warrants explicit explanation. WRES and CWRESI are computed by NONMEM from the marginal distribution of observations, using the full variance–covariance structure implied by the Y equation, OMEGA, and SIGMA [3].

For the additive model, the marginal residual variance entering the likelihood is determined solely by the SIGMA term appearing in the observation model Y. When the model is written as Y = IPRED + ERR(1), the residual variance is simply SIGMA(1,1), independently of the user-defined scaling factor W used to compute IWRES. Consequently, changing W from SQRT(SIGMA(1,1)) (ADD.1) to W = 1 (ADD.2) does not modify the statistical model used for estimation and, therefore, leaves WRES and CWRESI unchanged. This was confirmed numerically: the maximum absolute difference in CWRESI between ADD.1 and ADD.2 was ≤ 10^−4^, attributable to NONMEM’s output rounding.

This invariance breaks down for the combined model when moving from two EPS to one EPS. With two epsilon terms (Y = IPRED + IPRED*ERR(1) + ERR(2)), NONMEM handles residual variability through a variance structure that is not internally identical to that used for a single-epsilon coding of the form Y = IPRED + W*ERR(1), even when the marginal residual variance is numerically similar at the observation level. As a result, parameterizations that are nearly equivalent for IWRES may still differ for population-level residuals.

Under FOCE, CWRESI depends on the variance model propagated through NONMEM’s internal variance–covariance computations. The one-EPS and two-EPS formulations, therefore, do not enter the approximation in the same way. This explains why VAR.3 and COMB SD can remain highly concordant with VAR.1 at the level of IWRES, yet still produce detectable differences in CWRESI for individual observations.

### 4.5. Relationship with Proost (2017)

A key conceptual point is that VAR-based parameterizations represent the true variance of the combined error model, whereas the SD parameterization approximates the standard deviation as a linear function of IPRED. This approximation is generally adequate at moderate concentrations but may diverge at low concentrations.

The present results directly extend the analysis of Proost (2017) [9] in two directions. First, we numerically confirm that the equivalence of VAR.1 and VAR.3 parameterizations—demonstrated by Proost at the level of parameter estimates and OFV—extends to individual diagnostic residuals, as follows: IWRES_3_/IWRES_1_ ≈ 1.00 (SD = 0.003, Pearson r = 1.00). Second, we demonstrate that the COMB SD coding, identified by Proost as producing different but valid parameter estimates (OFV_S_^D^ = −1844.85 vs. OFV^γ^_ar_ = −1800.81, ΔOFV = 44.04), also produces detectably different WRES and CWRESI (maximum CWRESI difference = 0.577) and introduces local IWRES instability at low concentrations (Table 2 (Panel A)).

Importantly, the ΔOFV of 44.04 between COMB SD and COMB VAR.1 cannot be interpreted as evidence of better or worse model fit. As Proost (2017) [9] clarifies, the COMB SD parameterization does not model the same error distribution as the VAR methods, it models W as a linear function of IPRED rather than as the true standard deviation of the combined variance. These two models are therefore not nested and their OFV values cannot be directly compared for model selection. This distinction is critical in practice: a pharmacometrician applying likelihood-ratio testing between COMB SD and COMB VAR models would be committing a methodological error.

Unlike Proost (2017), who analyzed real-world data with unknown true parameters, our simulation-based approach allows assessment of parameter recovery.

The COMB SD parameterization estimated CV_prop = 18.0% and σ_add = 0.313 mg/L, both different from the true values (15% and 0.5 mg/L) and from the VAR estimates (CV_prop ≈ 29%, σ_add ≈ 0.384 mg/L). This underscores that COMB SD does not estimate exactly the same quantities as the VAR methods, even when applied to the same data. This difference should be interpreted as a consequence of parameterization rather than as evidence that the SD method is inherently inappropriate.

### 4.6. Practical Implications

Based on the results of this study, the following practical recommendations can be formulated:

- Always define W as the model-predicted standard deviation of the residual error when interpreting IWRES;
- Avoid comparing IWRES across model runs unless W is defined consistently;
- Do not use IWRES-based criteria (e.g., |IWRES| > 2) when W is not normalized;
- Prefer CWRESI or simulation-based diagnostics (e.g., VPCs) for model evaluation;
- For combined error models, prefer the two-EPS variance-based parameterization (VAR.1) when population-level diagnostics are of interest;
- Interpret extreme IWRES values in SD parameterizations with caution, especially at low predicted concentrations.

These recommendations aim to ensure consistent and interpretable use of residual diagnostics in NONMEM.

**IWRES plots require a normalized W**, but IWRES should be interpreted within the broader hierarchy of pharmacometric diagnostics. In current practice, CWRESI and simulation-based diagnostics such as VPCs or NPDE are generally more robust than IWRES for model evaluation, especially in nonlinear settings. Nevertheless, because IWRES remains widely used in NONMEM workflows, its correct scaling remains important. For IWRES to be interpretable as a standardized residual with unit variance, W must incorporate the estimated residual standard deviation. Non-normalized codings (W = 1, W = IPRED) produce IWRES compressed by a factor of √(SIGMA(1,1)) relative to correctly normalized runs. In our data, this compression reached a factor of 3.5 for the proportional model (SD(IWRESprop2) = 0.269 vs. 0.933 for PROP.1). Any numerical criterion applied to IWRES distributions, such as the proportion of observations with |IWRES| > 2, will be strongly distorted by non-normalized coding and should not be used without first verifying that W is correctly defined.

**WRES and CWRESI are robust to W coding for simple models.** This invariance is consistent with the broader observation that population-level residuals are more robust diagnostic tools than individual-level residuals in nonlinear mixed-effects models [6]. For additive and proportional error models, WRES and CWRESI are invariant to the W definition, as confirmed numerically to a precision of 10^−4^. Diagnostic plots based on CWRESI can, therefore, be reliably interpreted regardless of the W coding used, which is reassuring given its recommended role as a primary diagnostic for FOCE-estimated models [3,4].

**Cross-run IWRES comparisons are misleading unless W consistency is verified.** As illustrated in Section 3.5, comparing IWRES-based diagnostic plots between runs using different W definitions can generate entirely artifactual differences in apparent residual scatter. A non-normalized additive run (ADD.2, SD(IWRES) = 0.440) compared visually with a normalized proportional run (PROP.1, SD(IWRES) = 0.933) would falsely suggest that the proportional model has twice the residual variability, a conclusion with no basis in the underlying likelihood or fit quality.

**For the combined error model, prefer two SIGMA for population-level diagnostics.** The VAR.1 two-EPS parameterization produces CWRESI fully consistent with the combined variance structure. The one-EPS alternatives (VAR.3, COMB SD) introduce small but detectable CWRESI differences (up to 0.586 for individual observations). When the primary objective is model comparison via population-level diagnostics, VAR.1 is preferred. VAR.3 remains a valid and practical alternative, with near-perfect concordance with VAR.1 for IWRES and parameter estimates.

**Exercise caution with COMB SD at low concentrations**. The COMB SD parameterization generated extreme IWRES values for 19 observations (0.32%) at IPRED ≤ 1.94 mg/L. These values do not reflect model misspecification but rather a numerical artifact of the linear W approximation where it diverges from the true combined standard deviation. This does not imply that the SD parameterization is intrinsically invalid or unusable. In some practical settings, it may remain attractive because of parameterization preferences or model fitting considerations. However, its diagnostic consequences should be recognized, especially when interpreting IWRES at low concentrations or comparing residual-based diagnostics across coding approaches.

In practical NONMEM workflows, the impact of W coding is likely to be most visible when IWRES plots are compared across runs using different coding conventions, when proportional or combined residual error models are used, and when low predicted concentrations amplify the effect of approximation in SD-based combined parameterizations.

### 4.7. Limitations

Several limitations of this study merit acknowledgement. First, all analyses were performed on simulated data from a single one-compartment oral absorption model with dense sampling and no deliberate model misspecification. This simplified framework was chosen to isolate the effect of residual error coding on diagnostic residuals under controlled conditions. Although the algebraic role of W in the definition of IWRES is general, the magnitude and practical visibility of the effects described here may differ in more complex settings, including multi-compartment models, nonlinear kinetics, sparse sampling designs, or models with stronger covariate structure. This is particularly relevant for combined error models and for population-level residuals, whose numerical behavior may be more sensitive to model structure and estimation context.

Second, all analyses were performed in NONMEM 7.6 with FOCE-I. Other nonlinear mixed-effects platforms, such as Monolix, Phoenix NLME, Pumas, or Bayesian frameworks, implement residual error models and residual diagnostics differently. Accordingly, the present conclusions should not be extrapolated mechanically beyond NONMEM, and the exact behavior of IWRES, WRES, and CWRESI under alternative coding strategies may differ across software environments. Third, we did not evaluate the impact of W coding on simulation-based diagnostics such as visual predictive checks (VPCs) or normalized prediction distribution errors (NPDE), which are increasingly recommended as alternatives to IWRES-based plots [4]. Fourth, the THETA-based parameterizations (ADD.3, PROP.3, and COMB VAR.3) produced IWRES that were normalized but not numerically identical to their SIGMA-based counterparts (ADD.1, PROP.1, and COMB VAR.1), reflecting minor differences in the optimizer convergence path. Users should be aware that even between normalized codings, small numerical IWRES differences may arise.

In addition, the present simulations used 500 subjects to obtain stable and visually clear residual patterns. The qualitative conclusions regarding the mathematical role of W are not expected to depend on this exact sample size. However, with smaller datasets, such as 100 or 200 subjects, estimation uncertainty, shrinkage, and random variability may become more prominent, and the observed effects may, therefore, appear less stable or less cleanly separated. Finally, evaluation of these issues in real-world datasets would be a valuable extension of the present work. Such analyses could help determine how often these coding-related distortions materially affect diagnostic interpretation in routine applied modeling settings.

## 5. Conclusions

This study demonstrates that, within NONMEM, the coding of W in the $ERROR block can strongly influence the numerical values of IWRES without altering the likelihood, the objective function value (OFV), or the estimated model parameters. When the observation model Y remains unchanged, alternative definitions of W rescale the individual residuals used to compute IWRES. In such cases, differences in IWRES reflect differences in residual standardization rather than differences in model fit.

Conversely, when residual variability is parameterized directly within the definition of Y, alternative codings may correspond to different parameterizations of the same variance model. These parameterizations can preserve the likelihood while modifying the interpretation and numerical values of estimated residual error parameters.

These findings have practical implications for pharmacometric modeling within the NONMEM framework. First, when IWRES are intended to behave as standardized residual diagnostics, W should correspond to the model-predicted standard deviation of the residual error. Failure to define W accordingly can produce misleading IWRES distributions, even when the underlying model fit is identical. Second, apparent discrepancies in IWRES across alternative model codings should not be interpreted as evidence of model misspecification without first verifying whether the underlying observation model Y has actually changed.

More broadly, this work highlights the importance of clearly distinguishing between the statistical model used for estimation and the scaling used for residual diagnostics. Misinterpretation of this distinction may lead to incorrect conclusions about model adequacy when comparing alternative residual error codings.

In practice, W affects the scaling of residual diagnostics, whereas the likelihood is determined by the residual variance encoded in the observation model.

## Figures and Tables

**Table 1:** True simulation parameters. True parameter values used for stochastic simulation of the three population pharmacokinetic datasets. Simulations were performed using rxode2 (v5.0.1) in R (v4.5.1) with a fixed random seed (20240101). A one-compartment model with first-order oral absorption (ADVAN2 TRANS2 equivalent) was used throughout.

**Panel A. Structural Model and Between-Subject Variability Parameters (Common to All Datasets)**							
**Parameter**	**Description**			**Units**		**Value**	
TVCL	Typical apparent clearance			L/h		5.0	
TVV	Typical apparent volume of distribution			L		50.0	
KA	Absorption rate constant (fixed)			h^−1^		1.0	
ω^2^ CL	Between-subject variance on CL (log-normal)			—		0.09 (~30% CV)	
ω^2^ V	Between-subject variance on V (log-normal)			—		0.09 (~30% CV)	
**Panel B. Residual error parameters (true values used for simulation)**							
**Dataset**	**Parameter**	**Description**			**Units**		**True value**
***Additive dataset***							
—	σ_add_	Additive standard deviation			mg/L		0.5
***Proportional dataset***							
—	σ_prop_	Proportional coefficient of variation			fraction (CV)		0.20 (20%)
***Combined dataset*** *							
—	σ_prop_	Proportional standard deviation			fraction (CV)		0.15 (15%)
—	σ_add_	Additive standard deviation			mg/L		0.5
**Panel C. Simulation study design**							
**Study design item**			**Value**				
Number of subjects			500				
Dose			100 mg (single oral dose)				
Sampling times			0.25, 0.5, 1, 1.5, 2, 4, 6, 8, 10, 12, 18, 24 h				
Observations per subject			12				
Total observations per dataset			6000				
Total records per dataset (with doses)			6500				
Random seed			20240101				

* The combined dataset was simulated using two independent normally distributed error terms, DV = IPRED × (1 + σprop × ε1) + σadd × ε2, consistent with the two-epsilon VAR.1 structure of Proost (2017) [9]. Abbreviations: TVCL, typical apparent clearance; TVV, typical apparent volume of distribution; KA, absorption rate constant; BSV, between-subject variability; CV, coefficient of variation; ω^2^, variance of between-subject variability on the log scale; σadd, additive standard deviation; σprop, proportional coefficient of variation.

**Table 2:** Estimated residual error parameters and diagnostic residual statistics for the nine NONMEM estimation runs. All runs used FOCE-I. Standard errors in parentheses.

**Panel A—Final Parameter Estimates (Standard Errors in Parentheses)**									
**Model**	**OFV**		**CL (L/h)**		**V (L)**	**BSV CL (ω^2^)**	**BSV V (ω^2^)**	**RUV Proportional ^1^**	**RUV Additive ^1^**
**Additive dataset**									
ADD.1	−2095.25		4.75 (0.096)		49.82 (0.787)	0.093 (0.012)	0.087 (0.007)	—	0.216 (SD = 0.465)
ADD.2	−2095.25		4.75 (0.096)		49.82 (0.786)	0.093 (0.012)	0.087 (0.007)	—	0.216 (SD = 0.465)
ADD.3	−2095.25		4.75 (0.096)		49.82 (0.787)	0.093 (0.012)	0.087 (0.007)	—	0.465 mg/L
**Proportional dataset**									
PROP.1	−9622.81		4.72 (0.061)		34.79 (0.473)	0.074 (0.005)	0.087 (0.006)	0.083 (CV = 28.8%)	—
PROP.2	−9622.81		4.72 (0.061)		34.79 (0.473)	0.074 (0.005)	0.087 (0.006)	0.083 (CV = 2 8.8%)	—
PROP.3	−9622.81		4.72 (0.061)		34.79 (0.473)	0.074 (0.005)	0.087 (0.006)	0.288 (CV = 28.8%)	—
**Combined dataset**									
COMB VAR.1	−1800.81		4.68 (0.097)		51.86 (0.871)	0.115 (0.015)	0.0836 (0.008)	0.084 (CV = 29.0%)	0.148 (SD = 0.385)
COMB VAR.3	−1800.81		4.68 (0.097)		51.86 (0.871)	0.115 (0.015)	0.0836 (0.008)	0.290 (CV = 29.0%)	0.389 mg/L
COMB SD	−1844.85		4.67 (0.096)		51.74 (0.866)	0.119 (0.015)	0.0859 (0.009)	0.180 (CV = 18.0%)	0.314 mg/L
**Panel B—Diagnostic residual statistics (n = 6000 obs per dataset)**									
		**IWRES ^2^**				**CWRESI**		**WRES**	
**Model**		**Mean**		**SD**		**Mean**	**SD**	**Mean**	**SD**
**Additive dataset**									
ADD.1		0.006		0.947		0.013	1	0.041	1.009
ADD.2		0.003		0.440		0.013	1	0.041	1.009
ADD.3		0.005		0.947		0.013	1	0.041	1.009
**Proportional dataset**									
PROP.1		0.037		0.933		0.145	1.009	0.216	1.109
PROP.2		0.011		0.269		0.145	1.009	0.216	1.109
PROP.3		0.037		0.933		0.145	1.009	0.216	1.109
**Combined dataset**									
COMB VAR.1		0.027		0.946		0.024	1.003	0.053	1.01
COMB VAR.3		0.023		0.946		0.024	1.018	0.053	1.017
COMB SD		0.023		0.945		0.024	1.012	0.054	1.017

^1^ SIGMA-based (ADD.1/2, PROP.1/2, COMB VAR.1): SIGMA estimate with derived SD or CV. THETA-based (ADD.3, PROP.3, COMB VAR.3, COMB SD): THETA(4)/THETA(5) = residual SD/CV, SIGMA fixed to 1. ^2^ Expected SD(IWRES) ≈ 1.0 for a correctly specified normalized model; values < 1.0 reflect ε-shrinkage [5]. OFV for COMB SD is not directly comparable to COMB VAR methods [9]. True simulation parameters: CL = 5.0 L/h, V = 50.0 L, KA = 1.0 h^−1^ (fixed), ω^2^(CL) = ω^2^(V) = 0.09; additive σ = 0.5 mg/L; proportional CV = 20%; combined CV = 15%, σ = 0.5 mg/L. *Panel A* presents the final parameter estimates from each run: objective function value (OFV), typical clearance (CL) and volume of distribution (V) with their standard errors, residual error parameter estimates (expressed as SIGMA or THETA depending on coding), and between-subject variability estimates (ω^2^CL, ω^2^V). Runs sharing the same dataset and Y equation but differing only in W definition (ADD.1 vs. ADD.2; PROP.1 vs. PROP.2) produced identical OFV and parameter estimates confirming that, for these codings, changing the user-defined W without changing Y does not alter the likelihood function. *Panel B* presents the means and standard deviations of IWRES, CWRESI, and WRES across all 6000 observations for each run, together with the pairwise IWRES ratio relative to the normalized reference run within each error type (ADD.1, PROP.1, COMB VAR.1), and the Pearson correlation between IWRES vectors. The SD of IWRES quantifies the degree of normalization: a value of 1.0 indicates correct standardization under a well-specified model; departures reflect either non-normalized W coding or ε-shrinkage. *Abbreviations:* OFV, objective function value; CV, coefficient of variation; SD, standard deviation; IWRES, individual weighted residual; CWRESI, conditional weighted residual; WRES, weighted residual; ε-shrinkage, 1 − SD(IWRES) for normalized runs.

## Data Availability

The simulated datasets used in this analysis are available from the corresponding author upon reasonable request.

## Supplementary Materials

The following supporting information can be downloaded at: https://www.mdpi.com/article/10.3390/pharmaceutics18050590/s1, S1: Complete $ERROR and $SIGMA blocks for all nine NONMEM estimation runs; S2. R simulation script used to generate the three population PK datasets (additive, proportional, and combined) with rxode2.
