# Supplementary material for: Residual Error Coding in NONMEM Can Mislead Diagnostic Residuals: Impact of W Definition on IWRES, WRES, and CWRESI
Source: Pharmaceutics. 2026 May 10;18(5):590. doi: 10.3390/pharmaceutics18050590 (PMC13210693; doi:10.3390/pharmaceutics18050590)
Supplement: Supplementary file 1 [file pharmaceutics-18-00590-s001.zip › pharmaceutics-4226197-supplementary.pdf]

Supplementary Material

Residual error coding in NONMEM: impact on diagnostic residuals for additive, proportional, and combined error models

This Supplementary Material contains two sections:

S1. Complete $ERROR and $SIGMA blocks for all nine NONMEM estimation runs.

S2. R simulation script used to generate the three population PK datasets (additive, proportional, and combined) with rxode2.

All NONMEM runs used ADVAN2 TRANS2, METHOD=1 INTERACTION (FOCE-I), with KA fixed to 1.0 h^{-1}. The $PK, $THETA, $OMEGA, $ESTIMATION, and $TABLE blocks were identical across runs within each dataset and are not reproduced here. Only the $ERROR and $SIGMA blocks — which define the residual error model and differ between coding variants — are shown.

S1. NONMEM $ERROR and $SIGMA blocks for all nine estimation runs

S1.1 Additive residual error models (fitted to the additive dataset)

The additive dataset was simulated with σ_{aee} = 0.5 mg/L. All three coding variants produced identical OFV and parameter estimates.

ADD.1 — Normalized SIGMA-based coding (reference)

W is defined as the square root of SIGMA(1,1), which is the estimated residual variance. IWRES is therefore correctly standardized to unit variance under a well-specified model.

```
$ERROR
  IPRED = F
  IRES  = DV - IPRED
  W     = SQRT(SIGMA(1,1))       ; SD of additive error
  IF (W.EQ.0) W = 1              ; protection against W = 0
  IWRES = IRES / W
  Y     = IPRED + ERR(1)

$SIGMA
  0.25                           ; initial estimate; freely estimated
                                 ; SIGMA(1,1) = variance of additive error
```

ADD.2 — Non-normalized coding (W = 1)

W is fixed to 1, which is not the residual standard deviation. The Y equation is structurally identical to ADD.1, so the likelihood, OFV, and all parameter estimates are identical. Only IWRES is affected: it equals IRES rather than IRES/σ, and its standard deviation equals σ rather than 1.

```
$ERROR
 IPRED = F
 IRES  = DV - IPRED
 W     = 1                    ; non-normalized: W does not include SIGMA
 IWRES = IRES / W             ; = IRES (unnormalized)
 Y     = IPRED + W * ERR(1)     ; Y equation identical to ADD.1

$SIGMA
 0.25                         ; freely estimated; absorbs full residual variance
```

ADD.3 — THETA-based normalized coding ($SIGMA 1 FIX)

The residual standard deviation is estimated as a fixed effect THETA(4), with SIGMA fixed to 1. IWRES is correctly normalized provided THETA(4) converges to the true residual SD. This parameterization expresses the error magnitude on a clinically interpretable scale (mg/L).

```
$ERROR
 IPRED = F
 IRES  = DV - IPRED
 W     = THETA(4)             ; THETA(4) = additive SD (mg/L)
 IF (W.EQ.0) W = 1
 IWRES = IRES / W
 Y     = IPRED + W * ERR(1)

$THETA
 ...                          ; THETA(1)-THETA(3): structural PK parameters
 (0, 0.5)                     ; THETA(4): additive SD, initial estimate 0.5 mg/L

$SIGMA
 1 FIX                        ; fixed to 1; residual scale absorbed into THETA(4)
```

Note: THETA(4) in ADD.3 estimated the additive SD directly (converged value: 0.4652 mg/L), numerically equivalent to SQRT(SIGMA(1,1)) from ADD.1 (SQRT(0.4652) = 0.6820 — note SIGMA(1,1) in ADD.1 = 0.4652 is the variance, so SD = 0.6820 mg/L). Slight numerical differences between ADD.1 and ADD.3 in IWRES (SD: 0.645 vs 0.947) reflect different convergence paths on the residual error scale.

S1.2 Proportional residual error models (fitted to the proportional dataset)

The proportional dataset was simulated with CV = 20% (σ_{p}↜$_{op}$ = 0.20). PROP.1 and PROP.2 share an identical Y equation and produced rigorously identical IPRED values and parameter estimates.

PROP.1 — Normalized SIGMA-based coding (reference)

W correctly reflects the observation-specific residual standard deviation, which is proportional to IPRED. IWRES is standardized to unit variance.

```
$ERROR
  IPRED = F
  IRES  = DV - IPRED
  W     = SQRT(IPRED**2 * SIGMA(1,1))  ; = IPRED * SQRT(SIGMA(1,1))
  IF (W.EQ.0) W = 1
  IWRES = IRES / W
  Y     = IPRED + IPRED * ERR(1)       ; proportional error on IPRED

$SIGMA
  0.04                        ; initial estimate; SIGMA(1,1) = CV^2
```

PROP.2 — Non-normalized coding (W = IPRED)

W equals IPRED but omits SQRT(SIGMA(1,1)). The Y equation is identical to PROP.1; consequently IPRED, IRES, OFV, and all parameter estimates are identical. IWRES is compressed by the constant factor SQRT(SIGMA(1,1)) relative to PROP.1.

```
$ERROR
  IPRED = F
  IRES  = DV - IPRED
  W     = IPRED                  ; non-normalized: omits SQRT(SIGMA(1,1))
  IF (W.EQ.0) W = 1
  IWRES = IRES / W               ; = IRES/IPRED (unnormalized)
  Y     = IPRED + IPRED * ERR(1) ; Y equation identical to PROP.1

$SIGMA
  0.04                        ; freely estimated; identical to PROP.1
```

PROP.3 — THETA-based normalized coding ($SIGMA 1 FIX)

THETA(4) estimates the proportional coefficient of variation as a dimensionless fraction. W correctly scales IWRES to unit variance.

```
$ERROR
  IPRED = F
  IRES  = DV - IPRED
  W     = IPRED * THETA(4)       ; THETA(4) = CV fraction (e.g. 0.20 for 20%)
  IF (W.EQ.0) W = 1
  IWRES = IRES / W
  Y     = IPRED + W * ERR(1)

$THETA
  ...                       ; THETA(1)-THETA(3): structural PK parameters
  (0, 0.2)                  ; THETA(4): proportional CV, initial estimate 0.20

$SIGMA
  1 FIX
```

S1.3 Combined residual error models (fitted to the combined dataset)

The combined dataset was simulated with $\sigma_{p\text{↜}op}$ = 0.15 and σ_{aee} = 0.5 mg/L, using two independent EPS terms consistent with the VAR.1 structure. Nomenclature follows Proost (2017).

COMB VAR.1 — Two-EPS variance-based method (Proost VAR.1) — reference

W is defined as the square root of the total residual variance, incorporating both proportional and additive components. Two independent EPS terms are used, one for each variance component. This is the canonical combined error model in NONMEM and the recommended coding for population-level diagnostics.

```
$ERROR
  IPRED = F
  IRES  = DV - IPRED
  W     = SQRT(IPRED**2 * SIGMA(1,1) + SIGMA(2,2))
  IF (W.EQ.0) W = 1
  IWRES = IRES / W
  Y     = IPRED + IPRED * ERR(1) + ERR(2)  ; two EPS terms

$SIGMA
```

```
  0.04                  ; SIGMA(1,1) = proportional variance (CV^2)
  0.25                  ; SIGMA(2,2) = additive variance (SD^2)
```

COMB VAR.3 — One-EPS THETA-based variance method (Proost VAR.3)

Both proportional and additive standard deviations are estimated as THETA parameters, with SIGMA fixed to 1 and a single EPS term. W is constructed to equal the true combined residual standard deviation. This coding is equivalent to VAR.1 in terms of IWRES (r = 1.000) but produces slightly different CWRESI due to the one- versus two-EPS structure.

```
$ERROR
  IPRED = F
  IRES  = DV - IPRED
  W     = SQRT(IPRED**2 * THETA(4)**2 + THETA(5)**2)
  IF (W.EQ.0) W = 1
  IWRES = IRES / W
  Y     = IPRED + W * ERR(1)     ; single EPS term

$THETA
  ...                   ; THETA(1)-THETA(3): structural PK parameters
  (0, 0.15)             ; THETA(4): proportional SD (CV fraction)
  (0, 0.5)              ; THETA(5): additive SD (mg/L)

$SIGMA
  1 FIX
```

COMB SD — Standard deviation-based method (Proost SD)

W is expressed as a linear combination of the proportional and additive standard deviation components. This approximation does not equal the true combined standard deviation at the observation level (which is $SQRT(IPRED^2 \times \sigma^2 prop + \sigma^2 add)$), and produces different but statistically valid parameter estimates compared to VAR methods. As demonstrated in the main text, this coding introduces numerical instability in IWRES for observations with low predicted concentrations (IPRED ≤ 1.94 mg/L in this dataset).

```
$ERROR
  IPRED = F
  IRES  = DV - IPRED
  W     = THETA(4) * IPRED + THETA(5)  ; linear approximation of combined SD
  IF (W.EQ.0) W = 1
  IWRES = IRES / W
  Y     = IPRED + W * ERR(1)
```

```
$THETA
  ...                        ; THETA(1)-THETA(3): structural PK parameters
  (0, 0.15)                  ; THETA(4): proportional SD coefficient
  (0, 0.5)                   ; THETA(5): additive SD (mg/L)

$SIGMA
  1 FIX
```

Note: The SD method yields different parameter estimates from VAR methods (THETA(4) = 0.1801, THETA(5) = 0.3135 vs THETA(4) = 0.2899, THETA(5) = 0.3848 for VAR.3) and a different OFV (−1844.85 vs −1800.81 for VAR.1), consistent with the findings of Proost (2017). These differences do not indicate model misspecification but reflect the distinct parameterization of the residual error scale.

S2. R simulation script (rxode2)

The following R script was used to simulate the three population PK datasets used in this study. All datasets were generated with a fixed random seed (20240101) to ensure reproducibility. The script requires R ≥ 4.5.1 and rxode2 ≥ 5.0.1.

```
# ==============================================================
# Simulation of three population PK datasets
# Residual error coding paper — rxode2 v5.0.1, R v4.5.1
# Seed: 20240101
# ==============================================================

library(rxode2)
library(data.table)

set.seed(20240101)

# Structural model ----
mod <- rxode2({
  d/dt(depot) = -KA * depot
  d/dt(central) = KA * depot - (CL/V) * central
  IPRED = central / V
})

# True population parameters ----
TVCL <- 5.0    # L/h
TVV  <- 50.0   # L
KA   <- 1.0    # h^-1 (fixed)
omega_CL <- 0.09   # variance on log scale (~30% CV)
omega_V  <- 0.09

nsub  <- 500
times <- c(0.25, 0.5, 1, 1.5, 2, 4, 6, 8, 10, 12, 18, 24)
dose  <- 100   # mg

#  Simulate individual parameters ----
eta_CL <- rnorm(nsub, 0, sqrt(omega_CL))
eta_V  <- rnorm(nsub, 0, sqrt(omega_V))
CL_i   <- TVCL * exp(eta_CL)
V_i    <- TVV  * exp(eta_V)
```

```
# Function: simulate one dataset ----
simulate_dataset <- function(sigma_prop = 0, sigma_add = 0) {
  all_data <- vector('list', nsub)
  for (i in seq_len(nsub)) {
    ev <- eventTable()
    ev$add.dosing(dose = dose, nbr.doses = 1, dosing.to = 1)
    ev$add.sampling(times)
    params <- c(KA = KA, CL = CL_i[i], V = V_i[i])
    out    <- rxSolve(mod, params, ev)
    IPRED  <- out$IPRED
    eps1   <- rnorm(length(times), 0, 1)
    eps2   <- rnorm(length(times), 0, 1)
    DV     <- IPRED * (1 + sigma_prop * eps1) + sigma_add * eps2
    DV     <- pmax(DV, 0.001)   # truncate at 0.001 mg/L
    all_data[[i]] <- data.table(
      ID    = i,
      TIME  = times,
      DV    = DV,
      IPRED = IPRED,
      AMT   = 0,
      EVID  = 0,
      MDV   = 0
    )
  }
  # Add dose records
  doses <- data.table(
    ID = seq_len(nsub), TIME = 0, DV = 0,
    IPRED = 0, AMT = dose, EVID = 1, MDV = 1
  )
  rbind(doses, rbindlist(all_data))[order(ID, TIME)]
}

# Generate the three datasets ----
dat_add  <- simulate_dataset(sigma_prop = 0,    sigma_add = 0.5)
dat_prop <- simulate_dataset(sigma_prop = 0.20, sigma_add = 0)
dat_comb <- simulate_dataset(sigma_prop = 0.15, sigma_add = 0.5)

# Export as NONMEM-ready CSV ----
fwrite(dat_add,  'dataset_additive.csv')
```

```
fwrite(dat_prop, 'dataset_proportional.csv')
fwrite(dat_comb, 'dataset_combined.csv')

message('Datasets saved: ',
        nrow(dat_add[EVID==0]), ' observations per dataset,  ',
        uniqueN(dat_add$ID), ' subjects.')
```

Each output file contains 6,500 records per dataset: 500 dose records (EVID = 1, MDV = 1, AMT = 100) and 6,000 observation records (EVID = 0, MDV = 0, 12 time points × 500 subjects). The column IPRED in the exported files reflects the true model-predicted concentration and was not used in NONMEM estimation; it is retained for reference only.

Software versions: R 4.5.1, rxode2 5.0.1, data.table 1.15.4. All packages available from CRAN.
